# Health technology assessment of medicinal products in Greece: a 5-year (2018–2023) review of timelines and productivity

**DOI:** 10.1017/S0266462324000485

**Published:** 2024-11-04

**Authors:** Athanasios Chantzaras, Athanasios Margetis, Chara Kani, Vassilis Koutsiouris, Flora Bacopoulou

**Affiliations:** 1 Health Technology Assessment and Reimbursement Committee, Greek Ministry of Health, Athens, Greece; 2 School of Economics and Political Sciences, National and Kapodistrian University of Athens, Athens, Greece; 3 General Secretariat of Strategic Planning, Greek Ministry of Health, Athens, Greece; 4 Center for Adolescent Medicine and UNESCO Chair in Adolescent Health Care, First Department of Pediatrics, Medical School, National and Kapodistrian University of Athens, Aghia Sophia Children’s Hospital, Athens, Greece

**Keywords:** health technology assessment, HTA, reimbursement, health policy, medicines, access, Greece

## Abstract

**Objectives:**

To assess the health technology assessment (HTA) process in Greece from its establishment in 2018 until 2023 in terms of timeliness and productivity.

**Methods:**

Data were collected from the HTA Committee’s database and other publicly available sources. The overall study timeframe was divided into three periods: (i) July 2018–January 2020, (ii) January 2020–July 2021, and (iii) July 2021–February 2023.

**Results:**

During the study period, a total of 1,157 applications for medicinal products (MPs) (including 219 new active substances (NAS) and orphans) were submitted to the HTA Committee. The number of HTA recommendations increased from 60 (first period) to 641 (third period), while the backlog of MPs pending HTA and price negotiations decreased from 89 and 106 (January 2020) to 8 and 44 (February 2023), respectively. The median time intervals for all application types decreased significantly over time. In February 2023, the median time for clinical data assessment of NAS (excluding orphans) almost halved from 207 days in the first period to 114 days; median times for NAS and orphans from regulatory approval to HTA application were 420 and 457 days, and from HTA application to reimbursement 228 and 417 days, respectively.

**Conclusions:**

The performance of the HTA process in Greece improved significantly over time, with increased MP appraisals, backlog reduction, and decreased timelines. Delays in reimbursement of NAS were mainly caused by the long gap between regulatory approval and HTA application. Overall, HTA review times in Greece are now on par with that of well-established European HTA systems.

## Introduction

In recent years, the importance of health technology assessment (HTA) in supporting healthcare decision-making has increased, particularly as publicly funded health insurance plans strive to find transparent and sustainable models for financing healthcare (1). A growing number of countries worldwide are systematically applying HTA to optimize the allocation of their limited resources to new health technologies and interventions to achieve greater value for money spent (2). Greece is among the countries that have relatively recently introduced an HTA system in the decision-making for reimbursement of medicinal products (MPs) (3). This new framework was established in January 2018 and began operating in the summer of the same year (4–6). Following a decade-long economic depression, Greece still faces significant fiscal challenges and financial pressures on the healthcare system. Therefore, the new HTA framework presents an opportunity to ensure efficient allocation of the country’s scarce resources and to provide patients with access to innovative therapies at affordable prices and in a timely manner.

### The HTA Process in Greece: An Overview

This section outlines the fundamental components of the HTA process in Greece, structured according to the logical framework proposed by Bertram et al. (7).

#### Inputs

In January 2018, a significant overhaul of the reimbursement system was enacted (4), leading to the establishment of the Assessment and Reimbursement of Medicines for Human Use Committee, commonly referred to as the HTA Committee. Operating within the framework of the National Drug Organization (EOF) and under the oversight of the Minister of Health (MoH), its core responsibility is to assess medicines using HTA methodology and criteria, issuing recommendations to the MoH, the ultimate decision-making body. Comprising 11 members appointed by the MoH, the HTA Committee consists of experts in fields such as pharmacology, pharmacoepidemiology, or pharmacoeconomics (3;8). The HTA Committee receives support from external experts/assessors, selected from a certified EOF list as well as other university and scientific institutions (9). Additionally, legislative provisions stipulate that the HTA Committee should be aided by a Secretariat composed of 10 highly skilled civil servants (3), though the actual number of Secretariat employees often falls short of this requirement.

Another crucial body involved in the HTA process is the newly established Negotiation Committee, tasked with performing the economic evaluations and negotiating prices and discount rates for all pharmaceutical products (3;4). The Negotiation Committee operates under the supervision of the MoH and is based within the National Organisation for the Provision of Health Services (EOPYY) (4), the sole purchaser of healthcare services provided by the publicly funded National Health System (3). Comprising nine members, the Negotiation Committee consists of representatives appointed by the MoH, EOPYY, and EOF (4).

Stringent measures ensuring independence, objectivity, and transparency apply to members of both committees, external assessors, and Secretariat staff (4;6). Members of both committees are not employed on a full-time basis, and they serve a three-year term, renewable only once. In late 2019, both the HTA and negotiation committees underwent reshuffling following political changes earlier that year.

The Rules of Operations for the two committees were issued in summer 2018, coinciding with the effective start of the new HTA process (5;6).

#### Activities

Initially, the HTA process covered all types of MPs, including new active substances (NAS), new indications, new combinations, biosimilars, generics, and all therapeutic analogues of MPs for which an application had been submitted. However, in October 2019, a fast-track clinical data assessment process of 1 month was introduced for biosimilars and vaccines. Also, generics, fixed dose combinations, and certain other applications (e.g., packaging changes) were exempted from clinical data assessment altogether. Although these products are not subject to clinical assessment, they still undergo processing. This involves checking for compliance with the regulatory requirements of the Greek HTA system and includes (potential) budget impact assessment and price negotiations. The process concludes with a recommendation concerning their inclusion in the positive list. Furthermore, the provision regarding the assessment of therapeutic analogues was repealed (10). Additionally, products meeting predefined price limits (e.g., generics, biosimilars), were exempted from negotiation processes (10). The HTA Committee also has discretion to re-evaluate all medicines listed in the Positive Reimbursement List (4;6). Initially, the committee assessed individual patient requests for exceptional reimbursement of unlisted MPs, but a dedicated subcommittee took over this task in December 2020 (11;12).

Initially, MPs were required to meet the “external criterion” for assessment eligibility, which involved being marketed in nine Member States and reimbursed in two-thirds of them. Certain exemptions from these criteria were in place, such as for orphan drugs, vaccines, and biosimilars (4). However, a year later, these requirements were eased. Presently, an on-patent medicinal product can undergo assessment if reimbursed in at least five specified Member States out of 11 using HTA mechanisms. Exceptions were also introduced, notably for well-established use of medicines (10). Additionally, in October 2022, Greece incorporated a yearly horizon scanning process into the HTA mechanism, conducted by EOPYY and not part of the HTA Committee’s responsibilities (13).

The assessment process initiates with the submission of an application by the market authorization holder (MAH), comprising a comprehensive dossier and payment of the assessment fee (4;6). This dossier should contain essential documents, including regulatory documents, the European Public Assessment Report (if centrally approved), recommendations from other HTA bodies (if available), relevant studies, and a completed application form. The Ministry of Health has standardized the application form to ensure consistency and completeness across submissions. The application form covers various aspects such as product and disease description, clinical benefit (therapeutic added value, summary of clinical trials, innovation description), comparison with currently reimbursed options (competitor landscape, guidelines, treatment pathway), and economic evaluation, providing a thorough overview of the MP under assessment. Adaptation of cost-effectiveness and budget impact analyses to the Greek setting is imperative (4;6). This standardization is designed to minimize variability in the quality of submissions. However, despite these efforts, some variability still remains, particularly in the economic evaluations included in the dossiers. This is largely due to the absence of specific guidelines delineating the structure and methodology of the cost-effectiveness model and the lack of a defined Incremental Cost-Effectiveness Ratio (ICER) threshold as a decision criterion. Furthermore, the availability of local epidemiological and costing data is limited.

Once the application is submitted, the HTA Committee assigns a rapporteur from its members and may appoint external experts, up to a maximum of two, as needed to conduct the clinical data assessment (4;6;9;14). The clinical data assessment evaluates: (i) data quality, utilizing the GRADE criteria with methodologies specified in the Rules of Operation, (ii) added therapeutic value, categorized into four levels (major, significant, marginal, and non-quantifiable), with specific cut-offs defined by legislation, and (iii) innovation level, determined using the 5-level (A-E) Ahlqvist-Rastad rating system (4;6). The Greek framework bears resemblance to the German IQWIG approach (8).

MPs with a favorable clinical data assessment from the HTA Committee advance to the Negotiation Committee for economic evaluation and reimbursement pricing negotiations. Subsequently, the Negotiation Committee provides a reasoned opinion regarding the MPs’ budgetary impact (4;6). Negotiation details are not disclosed to the HTA Committee, which receives only a positive or negative opinion. Prioritization rules for the Negotiation Committee were introduced in October 2019, emphasizing applications with substantial budgetary implications or addressing unmet medical needs. Furthermore, the MoH gained the authority to refer certain medicines, especially those with high budget impacts, for price negotiations (10).

#### Output

The HTA Committee considers the opinion of the Negotiation Committee before making its final recommendation to the MoH, which holds the ultimate decision-making authority. Key assessment criteria include (4;6): (i) clinical benefit (severity and burden of disease, impact on mortality and morbidity, and safety and tolerability data), (ii) comparison with existing reimbursed treatments, (iii) reliability/robustness of clinical data, (iv) cost-effectiveness ratio, and (v) budget impact. Patients and scientific associations have the option to be invited or request participation in committee meetings to share their views (4;6), although such participation is not mandatory and is infrequent in practice.

The MAH is informed about the recommendation, but the report is not published due to unresolved legal issues regarding commercial confidentiality. Legislation mandates that the MoH issue a Ministerial Decision within 180 days of the application submission date. Although failure to meet this deadline implies rejection, this provision has never been invoked. In cases of rejection, the MAH may resubmit an application after 3 months. Following the ministerial decision, which incorporates any prescription restrictions set by the HTA Committee, mandatory prescription protocols are issued and integrated into the electronic prescribing system for MP reimbursement (4;6).

**Figure 1.**
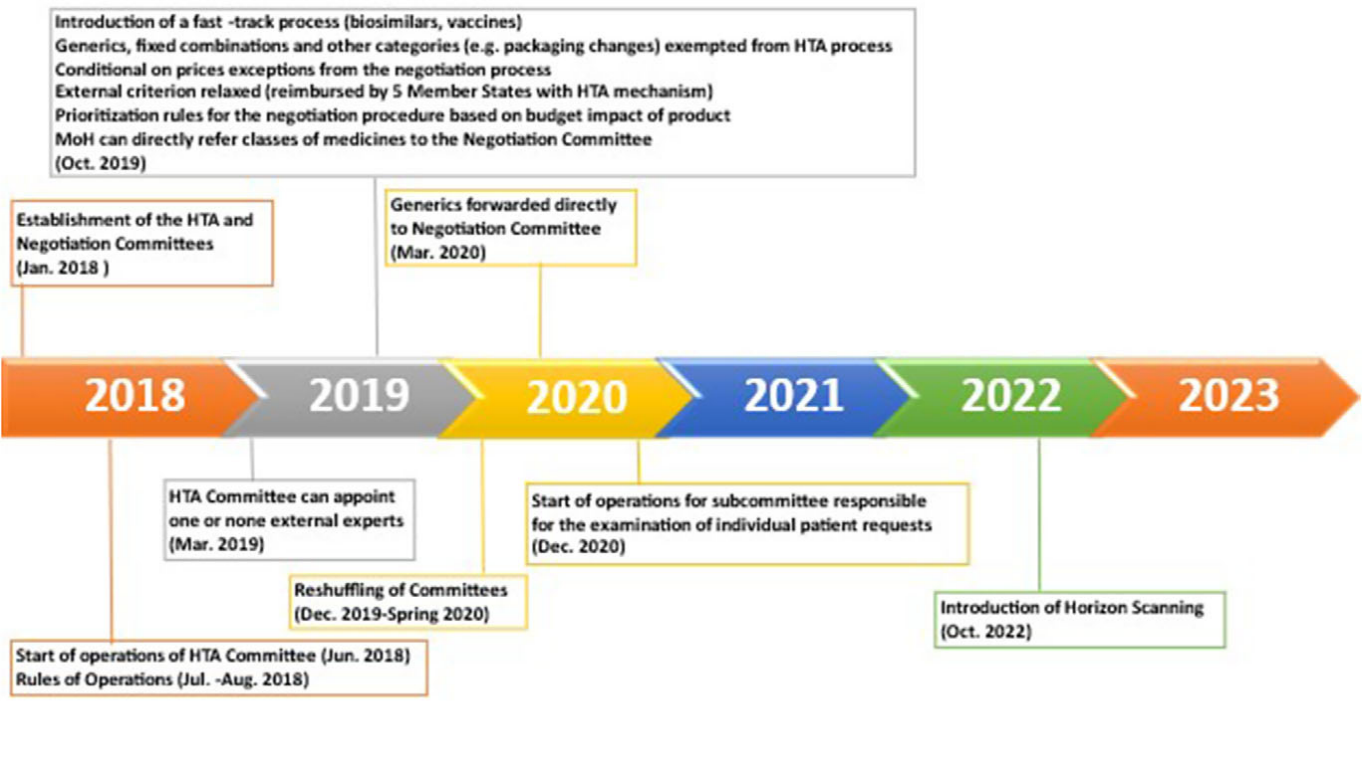
Key policy changes in the HTA process in Greece. HTA, health technology assessment; MAH, market authorization holder.

**Figure 2.**
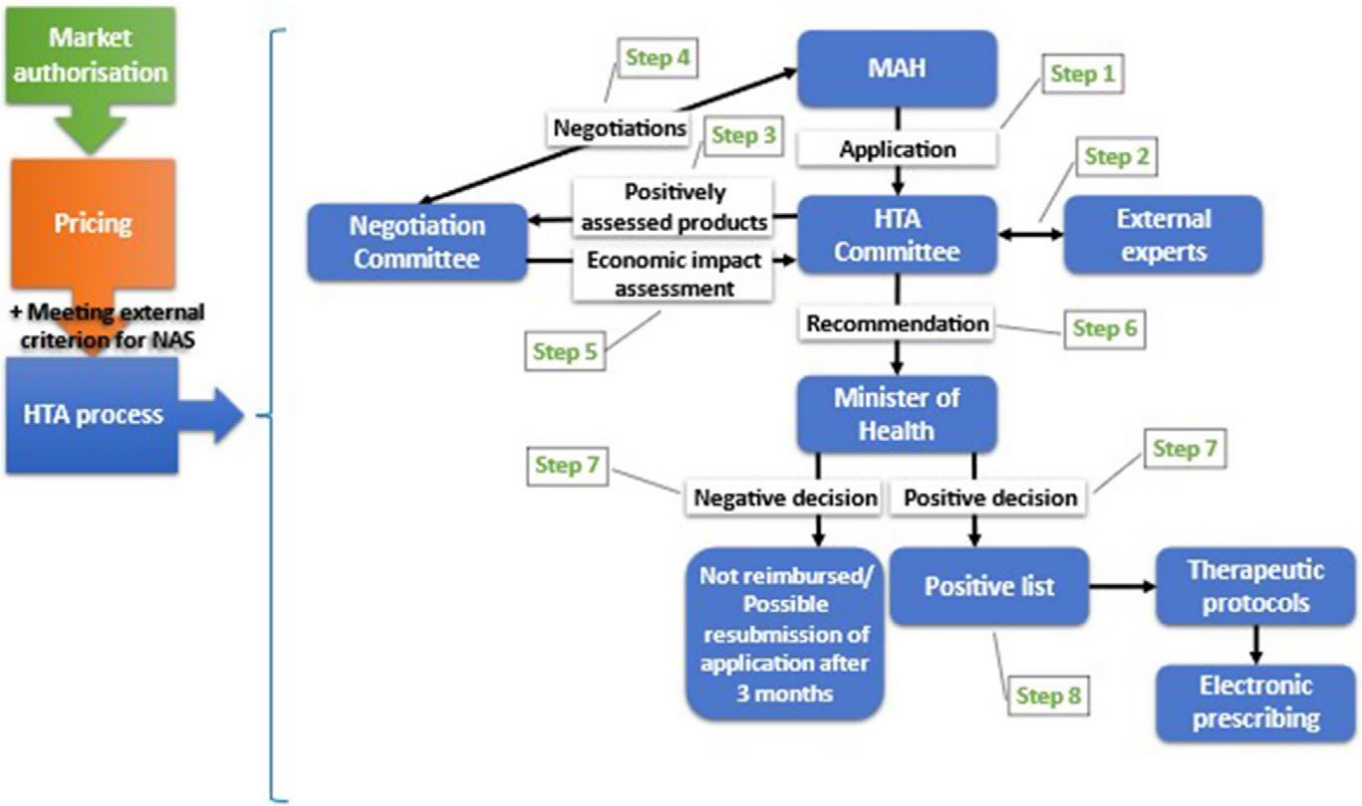
The current HTA pathway to reimbursement for NAS in Greece. HTA, health technology assessment; MAH, market authorization holder; NAS, new active substance.

Figure 1 illustrates the primary policy reforms within Greece’s HTA regulatory framework, while Figure 2 graphically depicts the current HTA pathway for reimbursement of NAS.

#### Outcomes

Although the Greek HTA framework includes a 180-day target for Ministerial Decision issuance and establishes specific timelines for certain procedures, it lacks statutory targets, indicators, and mechanisms for monitoring its progress over time. This study aims to address this gap by examining the performance of the HTA mechanism in Greece by utilizing quantitative indicators to assess aspects such as timeliness and productivity from its establishment until February 2023.

## Methods

All MPs for which an application was submitted to the HTA Committee from the enactment of the new HTA system in July 2018 to February 2023 were reviewed and included into the dataset. Data were collected from the HTA Committee’s database (not publicly available) as well as from other governmental (Ministerial Decisions, Positive Lists, Price Bulletins, and EOF) and nongovernmental (European Medicines Agency) public sources. Specifically, information was gathered on the legal basis and the type of market authorization procedure, the type of HTA application, therapeutic category according to the Anatomical Therapeutic Chemical (ATC) Classification System, direction of recommendation, and dates for specific milestones along the overall rollout timeline.

It should be noted that NAS not included in the Positive List were considered as new medicines. Additionally, some types of applications were grouped and analyzed together since they follow the same rules and procedures according to the current HTA regulatory framework in Greece (i.e., applications involving new pack-sizes with new containers and so on, new medicines with new indications (NM/NI), biosimilars with vaccines, known active substances with well-established use products and hybrids). In addition, a subset analysis was conducted to examine timelines for oncology MPs, which included antineoplastic and immunomodulating agents (classified under the ATC Classification System code: L).

The total time elapsed from market authorization to reimbursement decision was broken down into the time taken in calendar days for each step of the overall HTA process and by MP type:

- Time from the date of market authorization to the date of pricing (i.e., the date on which a new product, strength, pack-size and so forth is included in a Price Bulletin issued by the MoH).
- Time from the date of pricing to the date of HTA application. The date of the HTA application is defined as the date on which the application met the minimum requirements set by the legislation for an application to be valid for review. It does not consider any additional data that may be requested by the HTA Committee during the evaluation. If an application did not meet the minimum requirements, a clock-stop was applied.
- Time from the date of HTA application to the date of clinical data assessment (i.e., the date on which the clinical data assessment is finalized, and the product is referred to the Negotiation Committee or to the MoH in case of a positive or a negative opinion, respectively).
- Time from the date of clinical data assessment to the date of the Negotiation Committee’s opinion (i.e., the date on which the Negotiation Committee provides its opinion on the budget impact of the MP to the HTA Committee).
- Time from the date of the Negotiation Committee’s opinion to the date of recommendation (i.e., the date on which the HTA Committee renders its final recommendation to the MoH).
- Time from the date of recommendation to the date of ministerial decision (i.e., the date on which the MoH’s decision is issued).
- Time from the date of Ministerial Decision to the date of Positive List inclusion (i.e., the date of publication of a revised Positive List, which marks the date from which on a newly included product is reimbursed and/or an agreement between the Negotiation Committee and a MAH comes into effect).

During the study period, 11 revisions of the Positive List were released. Specifically, revisions were issued in (i) July 2019, (ii) January 2020, (iii) May 2020, (iv) July 2020, (v) January 2021, (vi) July 2021, (vii) February 2022 (twice in the same month), (viii) April 2022, (ix) November 2022, and (x) February 2023. These positive lists and their time points were used to create different analysis periods to evaluate the performance of the HTA system over time in terms of the time taken by different stages and the output (recommendations) produced. The overall study timeframe was divided into three observation periods, each lasting about one and a half years: (i) from July 2018 to January 2020, (ii) from January 2020 to July 2021, and (iii) from July 2021 to February 2023. Additionally, the outcomes of the latest Positive List released in February 2023 were also examined separately to provide the most updated review of the timeliness of the HTA system in Greece.

The results were summarized in reference to the observation period that encompasses the related Positive List, in which the MP was either included or not included, or removed due to a negative decision. In other words, starting from the listed products, time intervals were calculated for that specific basket of products. This method is relevant to assess the time needed for patient access to new therapies, and it enables international comparisons. We also computed time lags for aggregated stages of particular interest, such as the median time from the HTA application to the inclusion of the MP in the Positive List.

Results were summarized using descriptive statistics (median, interquartile range, frequencies). Statistical analysis was conducted using STATA v.17.

## Results

Between July 2018 and February 2023, a total of 1,157 applications were submitted to the HTA Committee, and an increasing trend was observed over time (Supplementary Table S1). Most of the applications concerned generics (*n* = 482) and NM/NI of NAS, including orphan drugs (*n* = 219). Of the 1,157 applications, 37.5 percent underwent the centralized procedure, 39.6 percent through decentralized or mutual recognition procedures, and the remaining 20.9 percent via national procedure (Supplementary Table S2). With regard to therapeutic indications, 26.4 percent of the MPs were antineoplastic/immunomodulating agents (Supplementary Table S3).

During the study period, a total of 1103 recommendations were issued, as indicated in Table 1. This figure also includes applications that did not undergo clinical data assessment or price negotiations. The number of recommendations increased significantly over time, from just 60 during the first study period (July 2018–January 2020) to 641 in the third period (July 2021 – February 2023). The number of recommendations issued for NM/NI applications, including orphan drugs, all of which underwent both clinical data assessment and price negotiations. Surged from a mere 4 during the first period to 53 and 135 in the subsequent two periods, respectively. In relation to the direction of recommendations, only 7 percent of them were negative, while 75.7 percent were positive, recommending full reimbursement of the products, and 17.3 percent recommended limited reimbursement for specific patient groups.

**Table 1.** HTA recommendations by observation period and type of MP application

Type of application	Direction of recommendation												
	July 2018 to January 2020				January 2020 to July 2021				July 2021 to February 2023				
	Neg.	Pos. w/restr.	Pos.	Total	Neg.	Pos. w/restr.	Pos.	Total	Neg.	Pos. w/restr.	Pos.	Total	Grand total
**NAS (excl. Orphan drugs)**													
New medicine/new indication	1	1	2	4	6	4	36	46	9	31	67	107	**157**
Other (e.g., new pack-size)	0	2	5	7	2	1	41	44	6	3	23	32	**83**
Re-evaluation	0	0	0	0	3	9	3	15	3	15	0	18	**33**
**Orphan drugs**													
New medicine/new indication	0	0	0	0	1	3	3	7	2	7	19	28	**35**
Other (e.g., new pack-size)	0	0	0	0	0	0	3	3	0	2	2	4	**7**
Re-evaluation	0	0	0	0	0	0	1	1	0	1	1	2	**3**
**Biosimilars/Vaccines**													
New medicine/new indication	0	0	8	8	0	4	21	25	2	7	8	17	**50**
Other (e.g., new pack-size)	0	0	0	0	0	0	4	4	1	0	2	3	**7**
Re-evaluation	0	0	0	0	1	0	5	6	0	1	1	2	**8**
**KAS/WEU/Hybrids**													
New medicine/new indication	0	0	1	1	7	4	13	24	9	23	52	84	**109**
Other (e.g., new pack-size)	0	0	0	0	1	0	16	17	2	13	23	38	**55**
Re-evaluation	0	0	0	0	0	0	0	0	5	36	0	41	**41**
**Fixed-dose combinations**	0	2	1	3	3	2	13	18	1	9	7	17	**38**
**Generics**	0	3	34	37	2	0	190	192	10	8	230	248	**477**
**Grand Total**	**1**	**8**	**51**	**60**	**26**	**27**	**349**	**402**	**50**	**156**	**435**	**641**	**1103**

*Note:* Despite varying in function and therapeutic characteristics, biosimilars are grouped with vaccines, and known active substances with well-established use and hybrid products. This grouping is due to their uniform processing under the Greek HTA system, where they adhere to identical regulatory procedures. The negative recommendations include 18 cases of withdrawal of application by the MAH. For another two applications, clock-stop has been implemented.

excl., excluding; HTA, health technology assessment; KAS, known active substance; MP, medicinal product; NAS, new active substance; neg., negative; pos., positive; w/restr., with restrictions; WEU, well-established use.

Between July 2018 and July 2021, 185 ministerial decisions were issued, including those revising the Positive List. The first Ministerial Decision, issued in late June 2019, covered 22 products, with 14 being generics. Notably, this was the only ministerial decision regarding new MPs until March 2020. It should be noted that there was alignment between the HTA Committee’s recommendations and the respective ministerial decisions.

In January 2020, 89 applications awaited clinical data assessment (HTA Committee’s backlog), while 106 were under reimbursement negotiations (Negotiation Committee’s backlog) (see Supplementary Figure S1). By February 2023, the number awaiting clinical evaluation decreased to 8, while the Negotiation Committee’s backlog reduced to 44. Supplementary Table S4 offers a detailed breakdown by MP application type.

Table 2 displays the median time in calendar days for each stage of the reimbursement process by MP application type and observation period. The median time interval between market authorization and HTA application submission was lengthy for all MPs but decreased since the initial analysis period. For NM/NI applications of NAS (excluding orphan drugs), the median time (25th–75th percentiles) for HTA application submission decreased from 568 (91–1246) median days during the first period to 459 (329–743) days between July 2021 and February 2023. Until January 2020, the median time for inclusion in a Positive List from the HTA application submission of NM/NI of NAS, excluding orphan drugs, was 342 (300–342) days, involving three MPs. For 39 MPs listed between January 2020 and July 2021, the time increased to 596 (332–825) days, then decreased to 394 (222–580) days for 98 MPs from July 2021 to February 2023. This inverse U-shaped trend, observed also with orphan MPs, stemmed from fluctuations in reimbursement price negotiation times, which initially increased from 105 (80–139) days to 373 (67–488) days and later decreased to 146 (48–308) days. Conversely, clinical data assessment durations saw continuous decline, with median times dropping from 207 (176–210) days to 121 (73–200) days across the three periods. Overall, time intervals decreased gradually for other MP applications as well.

**Table 2.** Median (25th–75th percentiles) time intervals (in days) for each stage of the HTA and reimbursement process by type of MP application

Part 1						
	NAS (excl. orphans)		Orphan drugs		KAS/WEU/Hybrids	
Stage by study period	NM/NI	Other	NM/NI	Other	NM/NI	Other
**Positive lists up to January 2020**	(*n* = 3)	(*n* = 0)	(*n* = 0)	(*n* = 0)	(*n* = 1)	(*n* = 0)
ΜΑ – Pricing	686 (267–1105)				240	
Pricing – HTA submission	221 (141–301)				141	
HTA submission – Clinical Data Assessment	207 (176–210)				316	
Clinical Data Assessment – NC opinion	105 (80–139)				4	
NC opinion – Recommendation	12 (0–12)				7	
Recommendation – MD	6 (4–6)				6	
MD – Positive List	9				9	
MA – HTA submission	568 (91–1246)				381	
HTA submission – Recommendation	327 (287–327)				327	
HTA submission – Positive list	342 (300–342)				342	
MA – Positive list	910 (391–1588)				723	
**Positive lists: January 2020–July 2021**	(*n* = 39)	(*n* = 49)	(*n* = 6)	(*n* = 3)	(*n* = 17)	(*n* = 16)
ΜΑ – Pricing	309 (210–541)		322 (210–575)		328 (233–439)	
Pricing – HTA submission	147 (100–398)		63 (0–121)		300 (100–476)	
HTA submission – Clinical Data Assessment	187 (96–229)	42 (22–138)	215 (195–315)	386 (32–519)	105 (94–158)	37 (6–202)
Clinical Data Assessment – NC opinion	373 (67–488)	258 (124–390)	425 (113–549)		90 (39–232)	194 (90–297)
NC opinion – Recommendation	15 (6–29)	15 (6–23)	14 (6–24)		13 (6–15)	9 (3–15)
Recommendation – MD	15 (7–21)	15 (6–27)	10 (7–13)	6 (6–20)	7 (6–22)	21 (6–24)
MD – Positive List	31 (17–84)	17 (4–66)	8 (8–8)	4 (4–91)	18 (17–31)	27 (4–97)
MA – HTA submission	434 (227–679)		345 (210–575)		730 (485–849)	
HTA submission – Recommendation	544 (251–728)	85 (35–288)	717 (665–764)	389 (42–536)	204 (165–356)	90 (18–251)
HTA submission – Positive list	596 (332–825)	171 (60–375)	735 (680–785)	399 (52–647)	268 (240–379)	175 (115–319)
MA – Positive list	1010 (827–1255)		956 (890–1228)		990 (816–1345)	
**Positive lists: July 2021–February 2023**	(*n* = 98)	(*n* = 26)	(*n* = 26)	(*n* = 4)	(*n* = 72)	(*n* = 35)
ΜΑ – Pricing	330 (230–571)		290 (221–828)		218 (153–422)	
Pricing – HTA submission	145 (115–294)		107 (52–178)		47 (21–94)	
HTA submission – Clinical Data Assessment	121 (73–200)	33 (4–60)	197 (70–261)	131 (78–242)	123 (77–182)	21 (12–103)
Clinical Data Assessment – NC opinion	146 (48–308)	63 (28–336)	317 (125–454)	198 (99–256)	99 (26–278)	32 (21–236)
NC opinion – Recommendation	10 (4–17)	15 (4–24)	12 (7–15)	12 (10–23)	8 (2–16)	13 (3–17)
Recommendation – MD	15 (11–19)	17 (13–19)	14 (8–19)	15 (13–18)	16 (11–19)	17 (14–21)
MD – Positive List	31 (14–73)	63 (32–125)	27 (11–61)	28 (24–58)	31 (18–73)	55 (17–85)
MA – HTA submission	459 (329–743)		407 (302–636)		291 (185–606)	
HTA submission – Recommendation	318 (183–503)	88 (14–384)	518 (363–623)	344 (306–402)	214 (163–462)	66 (38–226)
HTA submission – Positive list	394 (222–580)	180 (122–403)	576 (381–669)	383 (346–474)	291 (210–513)	166 (115–370)
MA – Positive list	908 (627–1239)		981 (826–1453)		586 (473–1177)	

Part 2				
		Biosimilars/Vaccines		
Stage by date of Positive List	Fixed-dose combinations	NM/NI	Other	Generics
**Positive lists up to January 2020**	(*n* = 3)	(*n* = 1)	(*n* = 0)	(*n* = 14)
ΜΑ – Pricing	233 (230–371)	903		239 (211–319)
Pricing – HTA submission	190 (106–300)	49		36 (17–72)
HTA submission – Clinical Data Assessment	125 (112–127)	60		216 (195–237)
Clinical Data Assessment – NC opinion	139 (86–141)	195		3 (3–17)
NC opinion – Recommendation	12	12		8 (4–12)
Recommendation – MD	6	6		6
MD – Positive List	9	9		9
MA – HTA submission	423 (336–671)	952		309 (232–439)
HTA submission – Recommendation	278 (210–278)	267		244 (203–285)
HTA submission – Positive list	293 (225–293)	282		259 (218–314)
MA – Positive list	716 (561–964)	1234		604 (472–708)
**Positive lists January 2020–July 2021**	(*n* = 15)	(*n* = 32)	(*n* = 4)	(*n* = 213)
ΜΑ – Pricing	219 (161–306)	353 (195–573)		204 (134–373)
Pricing – HTA submission	126 (12–434)	96 (47–209)		49 (13–154)
HTA submission – Clinical Data Assessment	127 (32–189)	128 (31–207)	48 (10–171)	234 (189–279)
Clinical Data Assessment – NC opinion	123 (69–195)	297 (216–321)	284 (284–284)	272 (96–364)
NC opinion – Recommendation	3 (2–15)	15	11	
Recommendation – MD	15 (6–21)	17 (6–34)	23 (15–26)	18 (7–27)
MD – Positive List	18 (4–66)	24 (4–66)	113 (61–138)	36 (16–67)
MA – HTA submission	345 (204–748)	562 (278–694)		335 (189–615)
HTA submission – Recommendation	191 (105–338)	197 (59–478)	146 (24–322)	95 (69–288)
HTA submission – Positive list	212 (204–380)	319 (141–498)	222 (160–425)	195 (111–409)
MA – Positive list	552 (416–1170)	789 (638–1070)		669 (352–986)
**Positive lists July 2021–February 2023**	(*n* = 16)	(*n* = 14)	(*n* = 2)	(*n* = 238)
ΜΑ – Pricing	195 (173–263)	282 (196–388)		138 (93–306)
Pricing – HTA submission	41 (23–80)	38 (6–117)		10 (3–41)
HTA submission – Clinical Data Assessment	41 (19–142)	58 (39–75)	99 (60–138)	63 (63–64)
Clinical Data Assessment – NC opinion	237 (83–419)	58 (24–210)	289 (289–289)	299 (276–304)
NC opinion – Recommendation	16 (8–17)	10 (2–14)	5	
Recommendation – MD	19 (14–22)	15 (9–17)	13 (11–15)	14 (11–21)
MD – Positive List	73 (16–116)	24 (10–61)	12 (7–16)	70 (17–100)
MA – HTA submission	280 (193–367)	362 (223–579)		171 (101–515)
HTA submission – Recommendation	288 (69–493)	144 (111–236)	248 (63–432)	43 (31–71)
HTA submission – Positive list	386 (210–556)	261 (166–290)	272 (81–463)	122 (75–223)
MA – Positive list	796 (605–898)	633 (389–943)		333 (217–673)

*Note:* Despite varying in function and therapeutic characteristics, biosimilars are grouped with vaccines, and known active substances with well-established use and hybrid products. This grouping is due to their uniform processing under the Greek HTA system, where they adhere to identical regulatory procedures. The date of HTA submission is defined as the date from which on the application was valid to be reviewed. The 25th and 75th percentiles are not presented in cases where the calculations were based on just one product or there was no variation. The median time lag between MA and HTA submission is not always equal to the simple sum of the median time intervals of the stages MA to pricing and pricing to HTA submission, since products with applications concerning extension of indication do not get repriced via Price Bulletins (the reimbursement prices are different from the prices included in the Price Bulletins).

excl., excluding; HTA, health technology assessment; KAS, known active substance; MA, market authorisation; MD, ministerial decision; NAS, new active substances; NC, Negotiation Committee; NM/NI, new medicine/new indication; WEU, well-established use.

Table 3 shows median time intervals for MPs included in the last Positive List of the study period (February 2023). The median time from market authorization to HTA application for NM/NI of NAS, excluding orphan products, was shorter than for orphan drugs and biosimilars/vaccines (420 vs. 457 vs. 439 median days, respectively). Other MP applications had a median time of around 200 days. The median time from HTA application to listing was 228 days for NAS, excluding orphan products, and 417 days for orphan drugs. Clinical data assessment durations were 114 (57–204) days for NAS, excluding orphan products, and 203 (161–249) days for orphan drugs. Reimbursement price negotiation times were 60 (21–123) days for NAS, excluding orphan products, and 170 (69–376) days for orphan drugs. After negotiation completion, it took a median of 14 days for the HTA Committee to issue the final recommendation to the MoH, followed by 11 days for the Minister’s decision and 17 days for the MP to be added to the Positive List. Oncology product time intervals were comparable to those for NAS, excluding orphan drugs.

**Table 3.** Median (25th–75th percentiles) time interval (in days) for each stage of the HTA and reimbursement process by type of application for MPs included in the Positive List of February 2023

Stage by period	NAS (incl. orphan) overall	NAS (incl. orphan) oncology	NAS (excl. orphan)	Orphan drugs	KAS/WEU/Hybrids	Biosimilars/Vaccines	Fixed-dose combinations	Generics
	New medicine/new indication							
**Positive list of February 2023**	(*n* = 53)	(*n* = 32)	(*n* = 40)	(*n* = 13)	(*n* = 10)	(*n* = 4)	(*n* = 4)	(*n* = 43)
ΜΑ – Pricing	243 (210–806)	274 (218–785)	237 (196–785)	248 (210–828)	153 (138–192)	196 (184–503)	169 (109–201)	140 (100–415)
Pricing – HTA submission	145 (58–244)	145 (52–236)	145 (96–253)	150 (4–214)	19 (10–94)	102 (76–488)	24 (13–79)	81 (1–369)
HTA submission – Clinical Data Assessment	155 (68–208)	114 (59–205)	114 (57–204)	203 (161–249)	176 (122–182)	53 (41–60)	121 (13–228)	
Clinical Data Assessment – NC opinion	69 (34–198)	72 (19–158)	60 (21–123)	170 (69–376)	40 (7–90)	127 (17–278)	328 (179–544)	
NC opinion – Recommendation	14 (8–16)	14 (7–16)	14 (8–16)	14 (13–15)	14 (8–33)	11 (8–14)	8 (8–11)	
Recommendation – MD	11 (3–14)	11 (3–14)	11 (3–14)	11 (3–14)	8 (3–11)	3 (3–9)	9 (3–18)	13 (9–21)
MD – Positive List	17 (10–31)	17 (10–31)	17 (10–31)	17 (10–31)	17 (10–31)	10 (10–14)	14 (10–36)	45 (17–80)
MA – HTA submission	432 (284–832)	442 (283–619)	420 (260–856)	457 (360–832)	179 (157–460)	439 (184–626)	193 (122–280)	209 (142–1117)
HTA submission – Recommendation	300 (189–434)	211 (178–404)	207 (168–404)	368 (326–545)	196 (195–277)	178 (83–333)	455 (417–565)	26 (21–37)
HTA submission – Positive list	342 (209–465)	233 (208–441)	228 (200–442)	417 (376–570)	232 (221–290)	200 (105–346)	500 (454–594)	109 (70–123)
MA – Positive list	816 (577–1243)	742 (515–1119)	666 (485–1215)	1032 (826–1635)	435 (378–1048)	685 (289–971)	796 (605–865)	326 (249–1187)

*Note:* Despite varying in function and therapeutic characteristics, biosimilars are grouped with vaccines, and known active substances with well-established use and hybrid products. This grouping is due to their uniform processing under the Greek HTA system, where they adhere to identical regulatory procedures. The date of HTA submission is defined as the date from which on the application was valid to be reviewed. The median time lag between MA and HTA submission is not always equal to the simple sum of the median time intervals of the stages MA to pricing and pricing to HTA submission, since products with applications concerning extension of indication do not get repriced via Price Bulletins (the reimbursement prices are different from the prices included in the Price Bulletins).

excl., excluding; HTA, health technology assessment; incl., including; KAS, known active substance; MA, market authorisation; MD, ministerial decision; NAS, new active substances; NC, Negotiation Committee; WEU, well-established use.

## Discussion

The empirical part of this study evaluated the performance of the HTA process in Greece in terms of timeliness and productivity. Unlike most international studies that rely on publicly accessible data and assess the time from market authorization to HTA recommendation (15), this study leveraged the HTA Committee’s database to analyze time intervals for each stage of the HTA process by MP application type. This approach offers deeper insights into the HTA process and identifies potential areas for enhancement.

Timely access to new effective technologies is one of the key objectives of all health systems. One critical aspect of the overall rollout time is the duration between regulatory approval and application submission to the HTA agencies. Variation in this application time gap across countries can be attributed to the submission strategies of MAHs and the specific characteristics of each HTA regulatory framework (15). Our study observed a decreasing trend in the submission time gap in Greece over time, although it remains relatively high. For NAS, excluding orphan drugs, included in the Positive List of February 2023, the median submission time gap was 420 days, while it was 457 days for orphan drugs. In a study by Wang et al. (15) covering the period of 2014–2018, the median submission time gap for NAS authorized via the centralized procedure was notably shorter in other countries: 7 days in England, 23 days in Italy, 29 days in France, 42 days in Germany, and 49 days in Spain. In Ireland, the average time from market authorization to application submission for preliminary rapid review was estimated at 59 median days (16).

HTA agencies utilize various tools to reduce the rollout time for MPs at the presubmission stage. For instance, Australia and Canada offer a parallel review process, allowing HTA applications before market authorization, while Germany and France conduct presubmission meetings for scientific advice (15). In contrast, Greece lacks provisions for early engagement between the HTA Committee and MAHs before or after application submission. Early dialogue can streamline the process by offering clarifications and guidance, particularly regarding local data requirements (15). Therefore, integrating early engagement and communication is crucial for enhancing the efficiency of the HTA process in Greece.

In Greece, MPs must be priced before undergoing HTA review. The pricing system entails the requirement for the MP to be priced in at least three other Eurozone Member States, reduced to two for orphan drugs (3;17). This procedure can delay the pricing of new MPs and, consequently, their HTA application submission. However, pricing primarily involves economic evaluation and does not affect clinical data assessment. Thus, allowing HTA application submission irrespective of pricing status could expedite the process. Moreover, the external reference pricing in Greece averages the two lowest Eurozone prices (3). However, such a pricing system may hamper the availability of medicines in low-priced countries due to launch delays or manufacturers’ strategies to avoid lower prices cascading from one country to another (18).

Furthermore, the external criterion remains a barrier to initiating the HTA process for new MPs. To address this, reducing the number of reimbursement countries required or removing the prerequisite for HTA application submission and clinical data assessment initiation until the criterion is abolished altogether could be beneficial. However, even for applications unaffected by this criterion, such as orphan medicines, biosimilars, and vaccines, there is a significant submission time gap, potentially due to pharmaceutical companies’ marketing and submission strategies in Greece. The regulatory framework of the pharmaceutical market in Greece may incentivize manufacturers to delay product launches and HTA submissions due to the country’s small commercial size, low average prices for on-patent products, and high rebate and clawback requirements (3).

The median time taken from HTA application submission to inclusion in the reimbursement list for NAS excluding orphan drugs was estimated at 228 days for the MPs included in the last Positive List of the study period. Although it appears that the performance of the HTA process in Greece declined midway through the study period and then rapidly improved, this time variability reflects the impact of a significant backlog that accumulated until the end of 2019. The fluctuation in the average review time was due to varying durations of price negotiations, whereas median clinical data assessment times decreased consistently over time. Nevertheless, by the end of the study, both clinical data assessment and negotiation times reached their minimum (114 and 60 median days, respectively). Additionally, 14 days were required for the final recommendation to the MoH, 11 days for the ministerial decision, and 17 days for MP listing. In Canada, clinical data assessment, negotiation, and listing took 236, 273, and 67 mean days, respectively, for all NM/NI applications evaluated between 2012 and 2016 (19).

Significant variations exist in the time taken for new therapies to gain reimbursement across different countries. In the study of Wang et al. (15), the median HTA review time for NAS varied from 174 (157 for oncology products) days in France to 358 (389 for oncology products) days in Italy. Another study of 12 selected oncology MPs revealed diverse timelines, ranging from 199 days in Germany and 227 days in France to 504 days in Italy and 713 days in Spain (20). It should be noted that HTA decisions do not necessarily correspond to market access in all countries, since some of them, like Greece, require a separate procedure for the reimbursement decision (20). In another study in Canada, the average time from market authorization to listing was 602 days for all MPs reviewed from 2012 to 2016 (19). Switzerland experienced an increase in the average time from regulatory approval of oncology MPs to inclusion in the reimbursement list, rising from 234 days in 2009 to 463 days in 2018 (21). Spain also saw an increase in the average time to reimbursement, from 230 days in 2008 to 431 days in 2013 (22). A study of patient access to orphan drugs in selected European countries found that the shortest average time from market authorization to reimbursement decision was observed in Italy and France (18.6 and 19.5 months, respectively), while the longest was in England and Wales (27.6 and 29.3 months, respectively) (23). Finally, according to the EFPIA Patients W.A.I.T. Indicator 2022 Survey, the average time to reimbursement for a cohort of 168 MPs approved by the European Medicines Agency in the period 2018–2021 was 674 days in Greece, while it ranged from 128 days in Germany to over 1,351 days in Malta (24).

The Positive List is typically revised two to three times a year, encompassing all relevant Ministerial Decisions. It serves as the effective date for financial agreements and new product reimbursements. Rollout times can be reduced by increasing the frequency of revising the reimbursement list to four times a year and in regular, scheduled updates.

According to Transparency Directive 89/105/EEC, procedures for pricing and reimbursement of new MPs should not exceed 180 days in total, including 90 days for the decision on pricing and 90 days for the decision on reimbursement (25). It is important to note that although the duration for MPs to undergo Greece’s HTA process and join the Positive List may not be optimal, patient access to new MPs is not necessarily hindered. Patients can submit individual requests for reimbursement of MPs not yet assessed or included in the Positive List via their physicians. These requests are typically processed within a week, with approximately 76 percent receiving approval for reimbursement. Rejections usually stem from medical reasons alone, without economic criteria being considered. Moreover, all products approved through individual requests receive full reimbursement, unlike other early access programs such as France’s ATU, which refunds only the final negotiated price (26). This creates an economic incentive for some companies to delay HTA application submissions, thus widening the submission time gap. Addressing these concerns, there is potential to overhaul the case-by-case system by either transforming it into or complementing it with a program or fund granting temporary reimbursement status for promising new therapies in cases of clear unmet need and for a limited duration.

In Greece, the HTA process suffers from low time predictability, evidenced by significant variability in time intervals for procedures. This variability is common in Europe, possibly due to uncertainties in product effectiveness or safety (20). Additional evidence requests may prolong HTA reviews, though this is rare in Greece. While there is a 180-day target for ministerial decision issuance, no specific timelines exist for the individual procedure process, although there are specific timelines for some tasks within each procedure. Specific timelines for clinical data assessment, price negotiation, and decision could enhance predictability. Moreover, accountability and transparency could be bolstered through annual progress reports on specific performance targets or a proper accountability framework.

During the five-year study period, around 66.1 percent of NAS applications, including orphan medicines, received positive recommendations for reimbursement as per their approved indications. Restricted reimbursement was recommended for 24 percent of applications, while 9.9 percent received a negative recommendation (Supplementary Table S5). Thus, despite some time delays and variability in Greece’s HTA process, the highly predictable outcome somewhat offsets these issues. In comparison, the study of Wang et al. (15) found that Germany (80.5 percent) and Italy (67.6 percent) had the highest proportion of positive recommendations without restrictions for NAS, while Australia (27 percent) and Canada (36.1 percent) had the lowest.

In December 2021, the European Parliament and the Council adopted Regulation (EU) 2021/2282 on HTA. This regulation aims to enhance cooperation among Member States by establishing a permanent EU-level framework for joint clinical data assessment, early scientific consultations, horizon scanning, and voluntary cooperation in nonmandatory areas. Member States retain responsibility for nonclinical data assessment, appraisal, and decision-making. These rules will take effect in early 2025, allowing time for related implementing and delegated acts, with an additional five-year transitional period for EU countries to fully adapt to the requirements of the Regulation. Undoubtedly, HTA systems with limited resources and capacity might benefit the most from the implementation of this new framework and especially from the provisioned timeframe of assessment (30 days after Commission Decision).

It is reasonable to assume that the required adaptation will result in a more streamlined HTA process in Greece, as some of the abovementioned issues will be resolved by the implementation of this Regulation. However, this also presents a unique opportunity and demand for the policymakers in Greece to strengthen the HTA system even further at both the organizational and human resource levels. Various avenues for enhancing Greece’s HTA process can be identified. Firstly, it is imperative to restructure the committees involved into independent entities with funding tied to performance targets. Additionally, increasing staffing levels to meet workload demands is crucial. It is also essential that committee members are full-time, permanent employees rather than relying on a pool of external experts, which fails to foster institutional memory and capacity building. HTA is still nascent in Greece, suggesting ample opportunity for capacity building. Activities such as improving resource availability, networking, knowledge transfer, collaborating with other HTA agencies, and skills development can bolster HTA agencies and stakeholders’ abilities to conduct and utilize HTA for decision-making (27). Furthermore, the recent introduction of horizon scanning can facilitate the timely identification of emerging technologies, aiding in planning and priority setting. Given the current lack of specific rules for prioritizing HTA applications, such scanning has the potential to improve transparency and standardization within the HTA process.

The legislative framework should further define the role of economic evaluations in HTA, including comprehensive guidelines for their execution and integration into decision-making. The current lack of specific economic criteria may prolong negotiation times and render outcomes unpredictable. Establishing detailed guidelines for economic evaluation would not only provide clear directives to submitters on organizing their economic data but also enhance the quality and comparability of submissions across diverse applications. This clarity is essential for ensuring that economic assessments are both rigorous and uniformly applied, thereby facilitating more streamlined and predictable decision-making processes. Additionally, the lack of disclosure regarding the details of price negotiations to the HTA Committee hinders considering trade-offs between clinical and nonclinical aspects during final recommendation preparation. Also, stakeholder involvement, including MAHs, clinicians, and patients, is crucial in both the early engagement phase and throughout the HTA process. It ensures relevance, credibility, and acceptability, ultimately supporting informed decision-making.

Practical obstacles, such as inadequate epidemiological and costing data, impede economic evaluations and delay patient access to therapies due to the resulting uncertainties during price negotiations (28). The lack of robust epidemiological data presents particular challenges for rare diseases, which might discourage manufacturers from introducing their products to markets with uncertain commercial viability. The current system of case-by-case examination of patient reimbursement requests for unlisted products represents a missed opportunity for real-world data collection. Consequently, addressing these issues requires the issuance of formal guidelines on economic modeling of products, establishing clearer economic decision-making criteria, and enhancing conditions for clinical and costing data development, collection, and sharing with all stakeholders.

## Conclusions

This study sheds light on the challenges within Greece’s HTA framework, particularly in terms of timeliness and productivity. Despite a slow start in its initial phase, the HTA process gained momentum over time by increasing appraisal rates, reducing backlogs, and decreasing rollout time intervals. It is interesting that delays in reimbursement of new MPs mainly stemmed from the long gap between regulatory approval and HTA application, whereas the median HTA review time in Greece was comparable with that of well-established European HTA systems. This achievement is promising, considering the relatively recent implementation of the HTA system in Greece. While policy reforms should leverage the strengths and successes of the current HTA framework, it is also essential to examine the mechanisms that promote timeliness, transparency, and predictability of HTA systems in other countries to enhance the performance of the HTA process in Greece.

## Supporting information

Chantzaras et al. supplementary material Chantzaras et al. supplementary material

## Supplementary material

The supplementary material for this article can be found at http://doi.org/10.1017/S0266462324000485.
