# Supplementary material for: Health technology assessment of medicinal products in Greece: a 5-year (2018–2023) review of timelines and productivity
Source: Int J Technol Assess Health Care. 2024 Nov 4;40(1):e40. doi: 10.1017/S0266462324000485 (PMC11563181; doi:10.1017/S0266462324000485)
Supplement: Chantzaras et al. supplementary material [file S0266462324000485sup001.docx]

**Health Technology Assessment of Medicinal Products in Greece:**

**A 5-Year (2018-2023)** **Review OF TIMELINES AND PRODUCTIVITY**

**Online supplementary Material**

This Appendix includes information referred to in the full version of the article

**Table S1** HTA applications by MP submission date period, and type of application

| **Type of application** | **July 2018 to Jan. 2020** | **Jan. 2020 to July 2021** | **July 2021 to Feb. 2023** | **Total** |
| --- | --- | --- | --- | --- |
| **NAS**  **(excl. orphan drugs)** |  |  |  |  |
| New medicine/new indication | 54 | 46 | 76 | **176** |
| Other (e.g. new pack-size) | 19 | 39 | 26 | **84** |
| Re-evaluation | 16 | 0 | 23 | **39** |
| **Orphan drugs** |  |  |  |  |
| New medicine/new indication | 10 | 15 | 18 | **43** |
| Other (e.g. new pack-size) | 2 | 4 | 1 | **7** |
| Re-evaluation | 1 | 0 | 2 | **3** |
| **Biosimilars/Vaccines** |  |  |  |  |
| New medicine/new indication | 21 | 15 | 14 | **50** |
| Other (e.g. new pack-size) | 1 | 5 | 1 | **7** |
| Re-evaluation | 6 | 0 | 2 | **8** |
| **KAS/WEU/Hybrids** |  |  |  |  |
| New medicine/new indication | 20 | 47 | 50 | **117** |
| Other (e.g. new pack-size) | 5 | 19 | 32 | **56** |
| Re-evaluation | 0 | 0 | 45 | **45** |
| **Fixed-dose combinations** | 12 | 15 | 13 | **40** |
| **Generics** | 90 | 169 | 223 | **482** |
| **Total** | **257** | **374** | **526** | **1157** |

Note: Despite varying in function and therapeutic characteristics, biosimilars are grouped with vaccines, and known active substances with well-established use and hybrid products. This grouping is due to their uniform processing under the Greek HTA system, where they adhere to identical regulatory procedures.

Abbreviations: HTA, health technology assessment; MP, medicinal product; excl., excluding; Feb., February; Jan., January; KAS, known active substance; NAS, new active substance; WEU, well-established use.

**Table S2** HTA applications by type of MP, market authorisation procedure, and study period

| **Type of application** | **July 2018 to January 2020** | | | **January 2020 to July 2021** | | | **July 2021 to February 2023** | | | **Total** | | |
| --- | --- | --- | --- | --- | --- | --- | --- | --- | --- | --- | --- | --- |
|  | **Centralised** | **DCP/MRP** | **National** | **Centralised** | **DCP/MRP** | **National** | **Centralised** | **DCP/MRP** | **National** | **Centralised** | **DCP/MRP** | **National** |
| **NAS (excl. orphan)** |  |  |  |  |  |  |  |  |  |  |  |  |
| New medicine/new indication | 52 | 2 | 0 | 45 | 1 | 0 | 75 | 1 | 0 | 172 | 4 | 0 |
| Other (e.g. new container, new pack-size) | 10 | 8 | 1 | 17 | 17 | 5 | 19 | 3 | 4 | 46 | 28 | 10 |
| Re-evaluation | 13 | 2 | 1 | 0 | 0 | 0 | 18 | 1 | 4 | 31 | 3 | 5 |
| **Orphan drugs** |  |  |  |  |  |  |  |  |  |  |  |  |
| New medicine/new indication | 10 | 0 | 0 | 15 | 0 | 0 | 18 | 0 | 0 | 43 | 0 | 0 |
| Other (e.g. new container, new pack-size) | 2 | 0 | 0 | 4 | 0 | 0 | 1 | 0 | 0 | 7 | 0 | 0 |
| Re-evaluation | 1 | 0 | 0 |  |  |  | 2 | 0 | 0 | 3 | 0 | 0 |
| **Biosimilars/Vaccines** |  |  |  |  |  |  |  |  |  |  |  |  |
| New medicine/new indication | 20 | 1 | 0 | 13 | 2 | 0 | 13 | 1 | 0 | 46 | 4 | 0 |
| Other (e.g. new container, new pack-size) | 1 | 0 | 0 | 5 | 0 | 0 | 1 | 0 | 0 | 7 | 0 | 0 |
| Re-evaluation | 6 | 0 | 0 |  |  |  | 2 | 0 | 0 | 8 | 0 | 0 |
| **KAS/WEU/Hybrids** |  |  |  |  |  |  |  |  |  |  |  |  |
| New medicine/new indication | 0 | 8 | 12 | 5 | 17 | 25 | 2 | 14 | 34 | 7 | 39 | 71 |
| Other (e.g. new container, new pack-size) | 0 | 4 | 1 | 1 | 12 | 6 | 2 | 11 | 19 | 3 | 27 | 26 |
| Re-evaluation | 0 | 0 | 0 | 0 | 0 | 0 | 0 | 18 | 27 | 0 | 18 | 27 |
| **Fixed-dose combinations** | 8 | 3 | 1 | 3 | 12 | 0 | 5 | 7 | 1 | 16 | 22 | 2 |
| **Generics** | 7 | 44 | 39 | 14 | 120 | 35 | 24 | 149 | 50 | 45 | 313 | 124 |
| **Total** | **130** | **72** | **55** | **122** | **181** | **71** | **182** | **205** | **139** | **434** | **458** | **265** |

Note: Despite varying in function and therapeutic characteristics, biosimilars are grouped with vaccines, and known active substances with well-established use and hybrid products. This grouping is due to their uniform processing under the Greek HTA system, where they adhere to identical regulatory procedures.

Abbreviations: HTA, health technology assessment; MP, medicinal product; DCP/MRP, Decentralized Procedure/ Mutual Recognition Procedure; excl., excluding; KAS, known active substance; NAS, new active substance; WEU, well-established use.

**Table S3** HTA applications by MPs legal basis and ATC 1 category (July 2018 to February 2023)

| **ATC 1 category** | **Legal basis** | | | | | | **Total** |
| --- | --- | --- | --- | --- | --- | --- | --- |
|  | **New active substance (excl. orphan drugs)** | **Orphan** | **Biosimilars/ vaccines** | **KAS/WEU/ hybrids** | **Fixed-dose combinations** | **Generics** |  |
| Alimentary tract and metabolism | 37 | 7 | 0 | 72 | 8 | 69 | **193** |
| Antiinfectives for systemic use | 22 | 2 | 14 | 0 | 1 | 66 | **105** |
| Antineoplastic and immunomodulating agents | 128 | 27 | 38 | 13 | 1 | 98 | **305** |
| Antiparasitic products, insecticides and repellents | 0 | 0 | 0 | 2 | 0 | 1 | **3** |
| Blood and blood forming organs | 30 | 6 | 12 | 39 | 1 | 24 | **112** |
| Cardiovascular system | 15 | 0 | 0 | 7 | 15 | 101 | **138** |
| Dermatologicals | 9 | 1 | 0 | 8 | 1 | 5 | **24** |
| Genito-urinary system and sex hormones | 4 | 0 | 0 | 5 | 0 | 16 | **25** |
| Musculo-skeletal system | 3 | 3 | 0 | 7 | 1 | 13 | **27** |
| Nervous system | 21 | 2 | 0 | 7 | 0 | 47 | **77** |
| Respiratory system | 9 | 2 | 0 | 10 | 12 | 7 | **40** |
| Sensory organs | 10 | 1 | 0 | 14 | 0 | 0 | **25** |
| Systemic hormonal preparations, excluding sex hormones and insulins | 4 | 2 | 1 | 29 | 0 | 20 | **56** |
| Others | 7 | 0 | 0 | 5 | 0 | 15 | **27** |

Note: Despite varying in function and therapeutic characteristics, biosimilars are grouped with vaccines, and known active substances with well-established use and hybrid products. This grouping is due to their uniform processing under the Greek HTA system, where they adhere to identical regulatory procedures.

Abbreviations: HTA, health technology assessment; MPs, medicinal products; ATC, Anatomical Therapeutic Chemical classification system; excl., excluding; KAS, known active substance; WEU, well-established use.

**Figure S1** HTA and Negotiation Committees’ MPs backlog over time

**
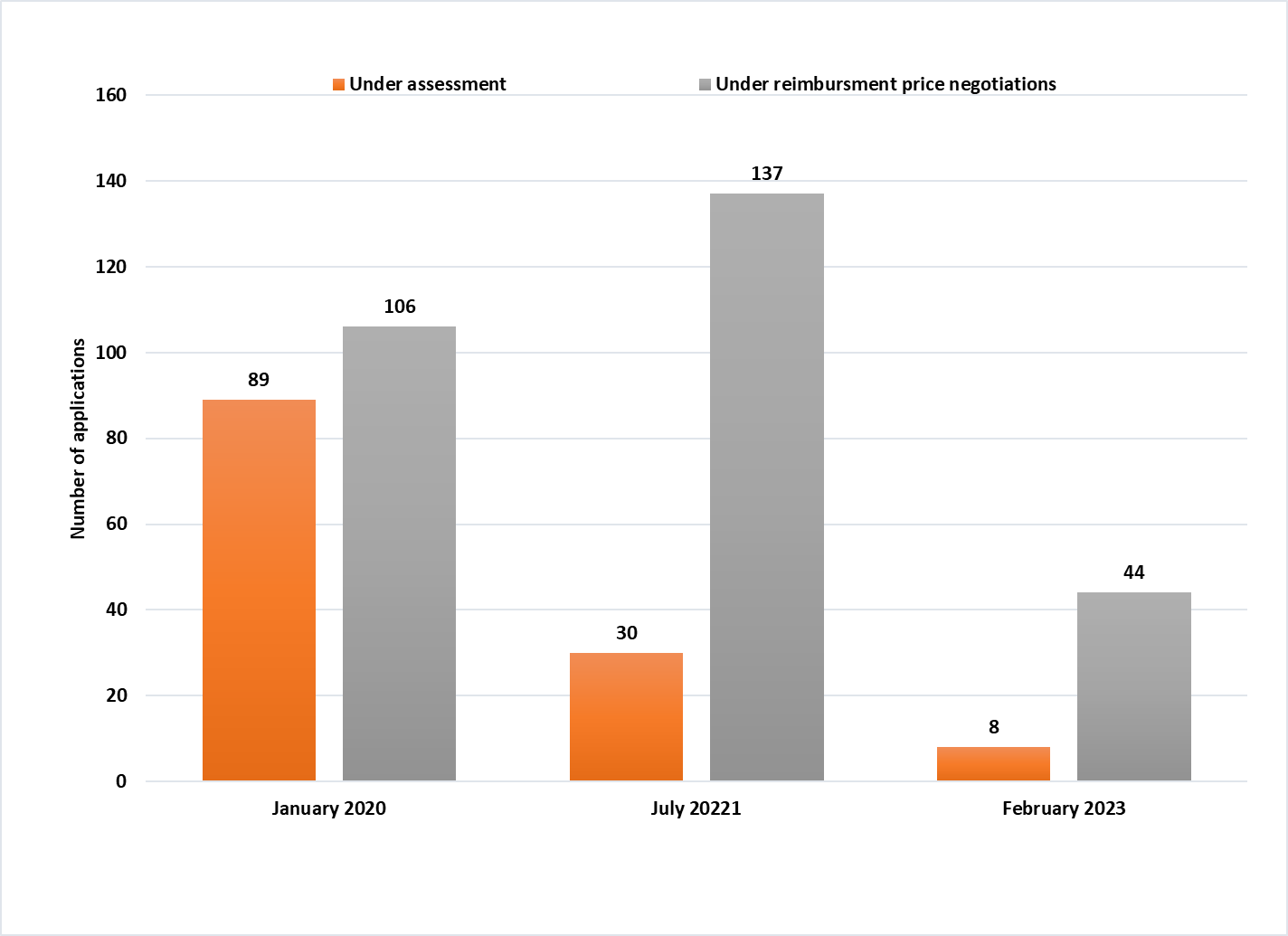
**

Abbreviations: HTA, health technology assessment; MPs, medicinal products

**Table S4** HTA and Negotiation Committees’ backlog by type of MP application

| **Type of application** | **January 2020** | | **July 2021** | | **February 2023** | |
| --- | --- | --- | --- | --- | --- | --- |
|  | **UA** | **UN** | **UA** | **UN** | **UA** | **UN** |
| **NAS (excl. orphan drugs)** | 26 | 23 | 14 | 36 | 4 | 14 |
| New medicine/new indication | 5 | 7 | 0 | 7 | 0 | 1 |
| Other (e.g. new container, new pack-size) | 8 | 8 | 0 | 1 | 0 | 6 |
| Re-evaluation |  |  |  |  |  |  |
| **Orphan drugs** | 4 | 6 | 3 | 15 | 1 | 7 |
| New medicine/new indication | 2 | 0 | 0 | 3 | 0 | 0 |
| Other (e.g. new container, new pack-size) | 0 | 1 | 0 | 0 | 0 | 0 |
| Re-evaluation |  |  |  |  |  |  |
| **Biosimilars/Vaccines** | 4 | 11 | 0 | 3 | 0 | 0 |
| New medicine/new indication | 0 | 1 | 0 | 2 | 0 | 0 |
| Other (e.g. new container, new pack-size) | 6 | 0 | 0 | 0 | 0 | 0 |
| Re-evaluation |  |  |  |  |  |  |
| **KAS/WEU/Hybrids** |  |  |  |  |  |  |
| New medicine/new indication | 13 | 6 | 10 | 32 | 3 | 5 |
| Other (e.g. new container, new pack-size) | 4 | 1 | 0 | 7 | 0 | 1 |
| Re-evaluation | 0 | 0 | 0 | 0 | 0 | 4 |
| **Fixed-dose combinations** | 3 | 5 | 2 | 3 | 0 | 1 |
| **Generics** | 14 | 37 | 1 | 29 | 0 | 5 |
| **Total** | 89 | 106 | 30 | 138 | 8 | 44 |

Note: Despite varying in function and therapeutic characteristics, biosimilars are grouped with vaccines, and known active substances with well-established use and hybrid products. This grouping is due to their uniform processing under the Greek HTA system, where they adhere to identical regulatory procedures.

Abbreviations: HTA, health technology assessment; MP, medicinal product; excl., excluding; KAS, known active substance; new active substances, NAS; UA, under clinical data assessment; UN, under negotiations; WEU, well-established use.

**Table S5** Recommendations per type of MP application (July 2018 - February 2023)

| **Recommendation**  **for Reimbursement** | **Type of MP application** | | | | | | |
| --- | --- | --- | --- | --- | --- | --- | --- |
|  | **NAS (incl. orphan)** | **NAS (excl. orphan)** | **Orphan drugs** | **Biosimilars/ vaccines** | **KAS/WEU/ Hybrids** | **Fixed-dose combinations** | **Generics** |
|  | **New medicine / new indication application** | | | | |  |  |
| **Negative** | 9.9% | 10.2% | 8.6% | 4.0% | 14.7% | 10.5% | 2.5% |
| **Positive with restrictions** | 24.0% | 22.9% | 28.6% | 22.0% | 24.8% | 34.2% | 2.3% |
| **Positive** | 66.1% | 66.9% | 62.9% | 74.0% | 60.6% | 55.3% | 95.2% |

Note: Despite varying in function and therapeutic characteristics, biosimilars are grouped with vaccines, and known active substances with well-established use and hybrid products. This grouping is due to their uniform processing under the Greek HTA system, where they adhere to identical regulatory procedures.

Abbreviations: MP, medicinal product; excl., excluding; incl., including; KAS, known active substance; NAS, new active substance; WEU, well-established use.
